# Acute psychosis with compulsive behaviour caused by hypothyroidism: a case report

**DOI:** 10.1192/j.eurpsy.2023.2242

**Published:** 2023-07-19

**Authors:** I. Ilgen Erdem, F. Alioglu Karayilan, B. Kalay Demirci

**Affiliations:** 1 Psychiatry, Karabuk University; 2 Psychiatry, Private Praxis, Karabuk, Türkiye

## Abstract

**Introduction:**

Hypothyroidism is a common problem in clinical practice which can be seen with diverse psychiatric manifestations.

**Objectives:**

Here we report a case of psychosis with compulsive behavior in a 46-year-old man who had no previous history of psychiatric disorder was brought to the emergency department by his family.

**Methods:**

During his evaluation, he described visual and auditory hallucinations. In addition, it was observed that dermatitis developed on her hands and around his mouth due to compulsive washing behavior. It was learned that he was diagnosed with hypothyroidism 2 months ago but he was not taking his medication. He was admitted inpatient clinic for 39 days and consulted to an endocrinologist. Inpatient treatment was initiated with haloperidol 10mg PO and levothyroxine. Given the persistence of irregular intake, it was decided to switch to haloperidol long-acting treatment.

**Results:**

After only requiring both antipsychotic drugs and thyroxine replacement our patient showed progressive clinical recovery attaining full remission within 5 weeks.

**Conclusions:**

It was clear that physicians should be aware of the possible different manifestations of endocrinologic disorders. All patients presenting with a first episode psychosis and compulsive behavior should be screened for thyroid dysfunction

**Disclosure of Interest:**

None Declared

